# An improved observer design approach for autonomous vehicles using error-based ultra-local model

**DOI:** 10.1038/s41598-025-10575-0

**Published:** 2025-07-11

**Authors:** Daniel Fenyes, Tamas Hegedus, Balazs Nemeth, Peter Gaspar

**Affiliations:** 1 HUN-REN Institute for Computer Science and Control (SZTAKI), Kende u. 13-17, 1111 Budapest, Hungary; 2 Department of Control for Transportation and Vehicle Systems, Budapest University of Technology and Economics, Stoczek u. 2, 1111 Budapest, Hungary

**Keywords:** Electrical and electronic engineering, Mechanical engineering

## Abstract

The paper presents a novel observer design method for an autonomous vehicle-oriented estimation problem. The design process combines two approaches: the Linear Parameter Varying framework and the error-based ultra-local model. The main goal of the error-based ultra-local model is to deal with the uncertainties and the nonlinearities of the model, whose effects cannot be taken into account during the modeling process. In this way, the performance of the LPV-based observer can be significantly improved. The proposed method is implemented for the estimation of the lateral velocity. The efficiency and the operation of the observer algorithm are presented through simulations in CarMaker and using real test measurements from ZalaZone proving ground.

## Introduction

The widespread of autonomous or highly automated vehicles requires a reliable and safe operation in every possible traffic scenarios. The complexity of the problem is well illustrated by the fact that numerous research institutes and industrial companies are dedicated to addressing autonomous vehicle-related challenges. Autonomous vehicles are complex systems, in which the algorithms, responsible for operation, can be divided into several layers considering functionality such as the sensing, decision-making, and control layer. For example, one of the main roles of the control layer is to guarantee accurate trajectory tracking, which is a primary requirement for safe operation. The performance level of the control algorithm highly relies on the accuracy of the measured signals. However, some of the states cannot be directly measured or the sensors are not affordable. Therefore, observer algorithms are used to estimate the states, which are essential for the control algorithm such as lateral velocity ($v_y$) or the side-slip angle ($\beta$).

Most of the observer methods, such as quadratic optimization^1,2^ or polytopic modeling (LPV)^3^, use a model of the considered system. One of the main advantages of these methods is that they have a theoretical guarantee of stability. In the paper^4^, a novel approach is introduced for estimation purposes of the side-slip angle and the lateral tire forces. During the estimation process, the variation of the tire stiffness is considered, which comes from the different tire-road friction coefficients and driving conditions. The LPV-based approach is applied for an optimal observer design, aiming to estimate vehicle parameters that cannot be directly measured^5^. In the paper^6^ proposes an observer design method for autonomous vehicles using the LPV-based approach, which aims to reconstruct the vehicle model, which depends on an online accessible time-varying parameter. Moreover, a damping force observer can be designed based on the LPV framework for a real automotive suspension^7^.

Another typical method for estimating the lateral velocity is the Kalman-filter-based solutions. In general, the Kalman filter algorithm is used to compensate for the drawback of a low-frequency GPS. For example, in^8^, an extended Kalman filter-based solution is presented using a nonlinear tire model. In^9^, a combined solution is proposed based on kinematic and dynamic models. A likelihood-based Kalman filter is proposed in^10^, which can deal with random measurement delays.^11^ presents a double cubature Kalman filter, which aims to optimize the covariance matrices. Although the Kalman-filter-based solutions provide good performance levels, there are some limitations: most of them need a GPS system, the covariance matrices should be known, and it is hard to deal with the nonlinear, unknown dynamics.

In recent years, the attention has shifted towards data-driven and machine learning-based solutions. The widespread of these techniques is motivated by their advantages compared to the model-based methods. Among the advantages, the capability of handling highly nonlinear systems can be highlighted. In papers^12,13^, neural network-based solutions are presented for side-slip angle and lateral velocity estimation for ground vehicles. Moreover, in^14^ an extensive comparison is given for side-slip estimation between model-based and data-driven-based solutions. The method is based on an extended and unscented Kalman filter, while the data-driven solution is a feed-forward and recurrent neural network-based solution. The results showed performance improvement using the data-driven solutions over the model-based approaches.

As presented, one of the main difficulties comes from the nonlinear dynamics of the vehicle. Even with modern techniques, such as LPV, hard to cover the whole operational range of the vehicle. Although the data-driven methods can provide better performance level, they are not reliable enough to implement in safety-critical systems, such as vehicles.

Thus, a new method has started to gain attention, which promises to solve the shortcomings of the model-based and data-driven methods. From a viewpoint of control design, it is known as the Model Free Controller (MFC)^15^. The main idea behind this approach is to approximate the dynamic of the real system by an ultra-local model (ULM). This ultra-local model is used as an additional control signal. The ultra-local control-based solutions have been successfully implemented for vehicle-oriented problems. In the paper,^16^ a ULM-based model predictive control is presented for lateral trajectory tracking of ground vehicles. In the paper^17^, an approach is presented using the ULM for lateral trajectory tracking in a decoupled framework considering various dynamic constraints and a wide range of longitudinal velocities. Moreover, an extension of the original structure is presented for the ULM in^18^. In the proposed method, not only the ultra-local model of the system but another ULM, based on a nominal model, is considered. Using these models, the error-based ultra-local model (EBULM) is composed, which aims to take into account the modeling mismatch between the real system and the nominal model. Moreover, a tuning method is proposed in^19^ for the extended ULM-based control structure. Although the original ULM has already been used for observer design purposes see^20,21^, the EBULM provides a new opportunity to improve the performance level of classical observers. The paper presents a novel method for estimating the unmeasurable state of a system, demonstrating this through the estimation problem of lateral velocity. The contribution of the paper is summarized:

- A novel application of the error-based ultra-local model for estimation problem of lateral velocity.
- The EBULM is combined with the LPV method to improve its performance level by eliminating the effects of unmodelled nonlinear dynamics.
- The proposed observer relies on only the available signal of the onboard system, meaning that no additional sensor is needed.
- The EBULM-based observer does not require information about the noises and the biases of the measured signals.
- The proposed observer method is almost data-free; the measurements are only needed to tune the free parameter of EBULM ($\alpha$). Thus, this observer is easy to implement and does not require high computational costs.

The proposed observer is validated through comprehensive simulations and using real test measurements. The simulations include tests on the surfaces with low adhesion coefficients and comparisons against baseline and neural network-based observers.

In the paper, the proposed algorithm is validated through real measurements using high-precision sensors. Moreover, the presented observer is tested under critical circumstances such as a low adhesion coefficient in a high-fidelity vehicle dynamics simulation software, CarMaker.

The paper is structured as: In “Observer design using ultra-local model” a brief introduction is given to the ultra-local model and the main steps of the observer design are presented. “Vehicle-oriented application” presents an application example of the proposed method, namely the observer design for the lateral velocity. The results of the proposed observer are then compared with real measurements. Moreover, the method is also tested in CarMaker simulation software to demonstrate its operation and efficiency, as detailed in “Validation of the proposed algorithm”. Finally, the contribution of the paper is summarized in “Conclusion”.

## Observer design using ultra-local model

In this section, a brief introduction is given to the original MFC structure and to the error-based ultra-local model. Then, the main steps of the combined observer design are detailed.

### Original MFC structure

The original Model Free Control algorithm was developed Fliess et al., see^15,22,23^. The main idea behind this concept is to model the nonlinear, unmodelled dynamics of the considered system by using an ultra-local model (*F*).

1a $$\begin{aligned} y^{(\nu )}&=F+ \alpha u, \end{aligned}$$

1b $$\begin{aligned} y^{(\nu )}_{ref}&=y^{(\nu )}_{ref}. \end{aligned}$$

This ultra-local model is derived from the input signal (*u*) and the $\nu ^{th}$ derivative of the measured output (*y*). Parameter $\alpha$ is a tuning variable of the control structure, which aims to scale the magnitude of the input to the output signal. Moreover, $y^{(\nu )}_{ref}$ is the derivative of the reference signal. The ultra-local model (*F*) can be calculated as:

2 $$\begin{aligned} F=y^{(\nu )}-\alpha u \end{aligned}$$

Using (1), the tracking error $e=y-y_{ref}$ obeys to the equation

3 $$\begin{aligned} e^{(\nu )}=y^{(\nu )}-y_{ref}^{(\nu )}=F+\alpha u-y_{ref}^{(\nu )}. \end{aligned}$$

Thus, $e^{(\nu )}=0$ can be achieved by the open loop control

4 $$\begin{aligned} u=\frac{-F+y_{ref}^{(\nu )}}{\alpha }. \end{aligned}$$

The computed *u* serves as an additional input signal for the system. In the case of any tracking problem, this control signal cannot guarantee the zero steady state error, therefore another feedback controller is needed (*C*(*s*)). Assuming that the ultra-local model (*F*) or its estimate ($F_{est}$) is available the control signal can be computed as:

5 $$\begin{aligned} u= \frac{-F_{est}+y^{(\nu )}_{ref}+C(s)e}{\alpha }, \end{aligned}$$

where, in practice, *C*(*s*) might be a PID^22^ or LQR^24^ controller.

### The error-based ultra-local model

The original structure, presented in the previous, has some implementation-related drawbacks as pointed out by^25^. Therefore, a new formulation has been introduced, which is called the error-based ultra-local model, see^26^. Basically, the error-based ultra-local is built up by two independent ultra-local models. The first ultra-local model uses the measured signals of the vehicle. The second one is constructed using a nominal model:

6a $$\begin{aligned} y^{(\nu )}&=F+\alpha u \end{aligned}$$

6b $$\begin{aligned} y^{(\nu )}_{ref}&=F_{nom}+\alpha u_{nom,ref} \end{aligned}$$

6c $$\begin{aligned} \underbrace{y^{(\nu )} - {y}^{(\nu )}_{ref}}_{{e}^{(\nu )}}&= \underbrace{F-F_{nom}}_{\Delta _{\text {nom}}} + \underbrace{\alpha u- \alpha u_{nom,ref}}_{\alpha \tilde{u}} \end{aligned}$$

6d $$\begin{aligned} e^{(\nu )}&=\Delta _{\text {nom}} + \alpha \tilde{u} \end{aligned}$$

$u_{nom,ref}$ is the reference input, while $y_{ref}$ is the reference output. *u* is the applied control signal and *y* is the measured output. Similarly to the original case, the additional control signal can be expressed as:

7 $$\begin{aligned} \tilde{u}_{EMPTY}= \frac{-\Delta _{\text {nom}}}{\alpha }, \end{aligned}$$

Note that in the original form (see:^18^), the control input is extended with an additional controller (*C*(*s*)). In that case, the applied control signal is computed as: $u_{\text {MFC}}= \frac{-\Delta _{\text {nom}}+C(s)e}{\alpha }$. However, in the case of observer design, this additional controller is neglected.

### LPV-based observer design

Since the goal of the paper is to present a combined observer design approach using LPV and ultra-local model-based techniques, a brief introduction is given to LPV systems and their observer design in the following subsection.

Let’s consider a nonlinear system, which is described by time-variant state matrices, see^27^:

8a $$\begin{aligned} \dot{x}&=A(\rho )x+B(\rho )u \end{aligned}$$

8b $$\begin{aligned} y&=C(\rho )x+D(\rho )\omega \end{aligned}$$

where $A(\rho )$, $B(\rho )$, $C(\rho )$, $D(\rho )$ are scheduling variable dependent matrices, *x* is the state-vector, *y* output of the system, *u* input signal, $\omega$ is the external disturbance, $\rho$ is the scheduling variable. Assume that the system is observable using one or more measured states of the system determined by state matrix $C(\rho )$. The goal of the observer design is to restore the non-measured states of the system using the measured signals and the control input (*u*) of the system. This means the minimization of the error between the real state-vector *x* and the estimated state-vector $\hat{x}$:

9 $$\begin{aligned} e=x-\hat{x}, \hspace{0.5cm} |e|\rightarrow min! \end{aligned}$$

state-vector $\hat{x}$ can be written as,^28^:

10 $$\begin{aligned} \dot{\hat{x}}=A(\rho )\hat{x}+B(\rho )u+L(\rho )(y-C(\rho )\hat{x}) \end{aligned}$$

the goal is to find the parameter dependent gain-vector $L(\rho )$, the observer gain. During the LPV formalism, various effects can be considered through the scheduling variable. However, in real systems, it is not always possible to fully cover the entire range of system parameters. Therefore, the goal is to use the results of the error-based ultra-local model, to consider these unmodeled dynamics or changing parameters:

11 $$\begin{aligned} \dot{\hat{x}}=A(\rho )\hat{x}+B(\rho )u+L(\rho )(y-C(\rho )\hat{x})-B_{\Delta }(\rho ) \frac{\Delta }{\alpha }, \end{aligned}$$

which means that the estimation is compensated through the term $B_{\Delta }(\rho )$. In the modeling process, $\Delta$ is handled as an additional contorl signal to the polytopic system, which compansate the unmodelled/nonlinear dynamics of the system by minimizing the derivative of the outputs of the nominal and real measured system ($y^{\nu }-\hat{y}^{\nu }\rightarrow min!$):

12 $$\begin{aligned} \dot{\hat{x}}=A(\rho )\hat{x}+B(\rho )(u- \frac{\Delta }{\alpha } )+L(\rho )(y-C(\rho )\hat{x}) \end{aligned}$$

Using (9) and (12):

13 $$\begin{aligned} \dot{e}(t) = (A(\rho ) + L(\rho ) C(\rho )) e(t) + B(\rho ) \frac{\Delta }{\alpha }. \end{aligned}$$

For stability, it must be guaranteeed that $e(t) \rightarrow 0$ and $\Delta (t) \rightarrow 0$ as $t \rightarrow \infty$. Using a quadratic Lyapunov function:

14 $$\begin{aligned} V(e) = e^T P e, \quad P > 0. \end{aligned}$$

Computing the time derivative of (14) $\dot{V}(e)=\dot{e}^TPe+{e}^TP\dot{e}$:

15 $$\begin{aligned} \dot{V}(e)&= e^T (A(\rho ) - L(\rho ) C(\rho ))^T P e+ e^T P (A(\rho ) - L(\rho ) C(\rho )) e + 2 e^T P B(\rho ) \Delta . \end{aligned}$$

To guarantee stability under varying $\rho$, the goal is to design $L(\rho )$ using Linear Matrix Inequalities (LMIs)^29^:

16 $$\begin{aligned} \begin{bmatrix} (A(\rho ) - L(\rho ) C(\rho ))^T P + P (A(\rho ) - L(\rho ) C(\rho )) & P B(\rho ) \\ B(\rho )^T P & -\gamma I \end{bmatrix} < 0. \end{aligned}$$

where $\gamma > 0$. Note that $\Delta$ must be limited $|\Delta |<\Delta _{max}$. In practice the optimization problem is solved by the LPV Toolbox^30^.

### The main steps of the observer design

The combined observer design consists of the following main steps:

1. Determination of the nominal model(s). (“Determination of the nominal model”)
2. Selection of the input, output singals and its derivative order ($\nu$). (“Selection of derivative order and computation of the reference signal”)
3. Computation of the nominal reference signals ($u_{nom,ref}$,$y^{\nu }_{ref}$ ) (“Selection of derivative order and computation of the reference signal”)
4. Design of LPV observer based on the nominal model.
5. Tuning of the parameter $\alpha$. (“Tuning the parameter α”)

Note that LPV observer must be discretized or designed in discrete form before the implementation, see^31^.

**Fig. 1:**
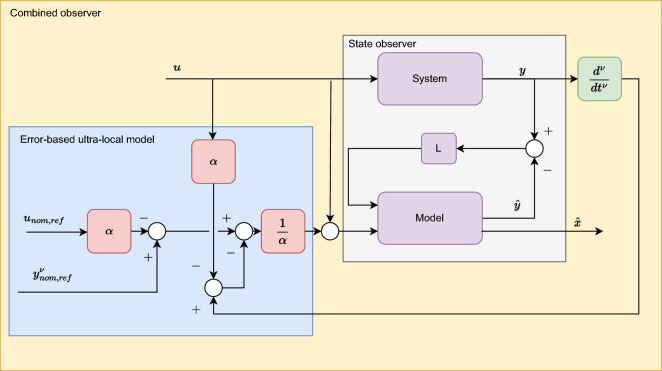
The structure of the proposed observer.

The overall structure of the observer is illustrated in Figure 1. The computation of specific signals is demonstrated through an example, with which the entire observer algorithm is validated.

## Vehicle-oriented application

This section outlines the main design steps of the combined observer algorithm for the vehicle-oriented problem, specifically focusing on the estimation of lateral velocity.

### Determination of the nominal model

As indicated in the description of the main steps in “The main steps of the observer design”, the first step is the selection of the nominal model. The lateral dynamics of the vehicle can be described by the two-wheeled bicycle model, see^32^. The bicycle model consists of two main equations: the first one describes the yaw motion of the vehicle while the second one models the lateral acceleration of the vehicle:

17a $$\begin{aligned} \ddot{\psi }I_z&=C_f(\delta -\beta -\frac{\dot{\psi }l_f}{v_x})l_f-C_r(-\beta +\frac{\dot{\psi }l_r}{v_x})l_r, \end{aligned}$$

17b $$\begin{aligned} a_ym&=C_f(\delta -\beta -\frac{\dot{\psi }l_f}{v_x})+C_r(-\beta +\frac{\dot{\psi }}{v_x}), \end{aligned}$$

17c $$\begin{aligned} a_y&=\ddot{y}_v+v_x\dot{\psi }, \end{aligned}$$

where $l_{f,r}$ geometric parameters of the car, $\dot{\psi }$ is the yaw-rate, *m* denotes the mass, $I_z$ is the yaw-inertia, $C_{f,r}$ are the cornering stiffness, $\beta$ is the side-slip, $v_{x,y}$ are the lateral and longitudinal velocities of the vehicle. The third equation describes the relation between the lateral acceleration ($a_y$) and the yaw-rate, while $y_v$ describes the translational motion of the vehicle in its coordinate system. Note that some of the parameters are not constant e.g. cornering stiffness or can change during the operation of the vehicle e.g. mass or the inertia.

#### State-space representation of the nominal model

The presented lateral vehicle model can be transformed into a parameter-dependent state-space representation. The scheduling parameter of the system is the longitudinal velocity ($v_x$)

18 $$\begin{aligned} \dot{x}=&A_v(\rho )x+B_v(\rho )u_v \end{aligned}$$

19 $$\begin{aligned} y_v=&C_v^T(\rho )x \end{aligned}$$

20 $$\begin{aligned}&\begin{bmatrix} \ddot{y}_v \\ \ddot{\psi } \end{bmatrix} = \underbrace{ \begin{bmatrix} -\frac{C_f+C_r}{m v_x} & \frac{C_f l_f - C_r l_r}{m v_x}-v_x \\ -\frac{C_f l_f - C_r l_r }{I_z v_x} & -\frac{C_f l_f^2 + C_r l_r^2}{I_z v_x} \\ \end{bmatrix} }_{A_v} \begin{bmatrix} \dot{y}_v \\ \dot{\psi } \end{bmatrix} + \underbrace{ \begin{bmatrix} \frac{C_f}{m} \\ \frac{C_f l_f}{I_z} \end{bmatrix} }_{B_v} \delta \end{aligned}$$

where $C^T_v=[1 \quad 0]$ and the scheduling parameter is the longitudinal velocity ($\rho =v_x$).

### Selection of derivative order and computation of the reference signal

In this paper, the goal is to observe the lateral velocity of the vehicle using the combination of the LPV and the error-based ultra-local model approaches. The lateral acceleration is a measurable signal, therefore, the order of the derivative is chosen to $\nu =1$. After discretization of the system (18), the following state space representation can be formed:

21 $$\begin{aligned} \begin{bmatrix} v_y(k+1) \\ \dot{\psi }(k+1) \end{bmatrix} = \underbrace{ \begin{bmatrix} a_{11} & a_{12} \\ a_{21} & a_{22} \\ \end{bmatrix} }_{A_v} \begin{bmatrix} v_y(k) \\ \dot{\psi }(k) \end{bmatrix} + \begin{bmatrix} b_1 \\ b_2 \end{bmatrix} \delta (k) \end{aligned}$$

### $u_{nom,ref}$ computation

**Fig. 2:**
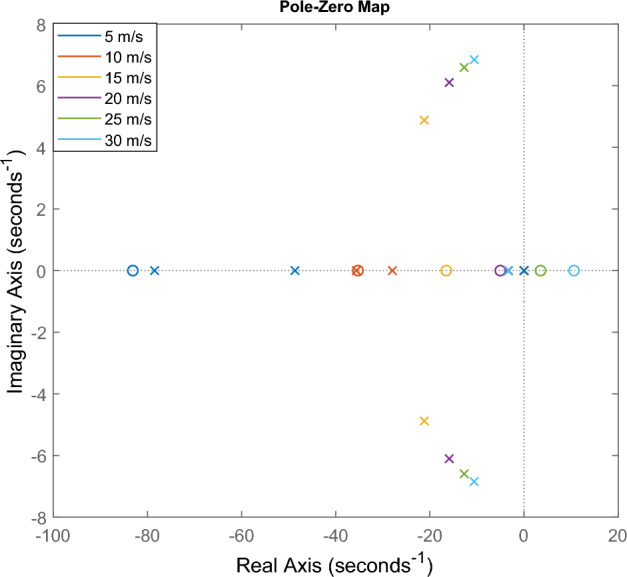
The poles and the zeros of the system.

The nominal control input ($u_{nom,ref}$) can be computed using the measured signals and the nominal model of the system. Although the nominal control input could be calculated using the inverse of the nominal model, stability-related issues arise in this application. In Figure 2 the pole-zero map can be seen, which shows that the inverse system becomes unstable at high longitudinal velocities. In the figure, crosses represent the poles, while circles indicate the zeros.

The lateral acceleration of the nominal model can be computed using the following state space representation:

22 $$\begin{aligned} \begin{bmatrix} \hat{v}_y(k+1) \\ \dot{\psi }(k+1) \\ \hat{v}_y(k) \end{bmatrix} = \underbrace{ \begin{bmatrix} a_{11} & a_{12} & 0 \\ a_{21} & a_{22} & 0 \\ 1& 0& 0 \end{bmatrix} }_{\Phi } \underbrace{ \begin{bmatrix} \hat{v}_y(k) \\ \dot{\psi }(k) \\ \hat{v}_y(k-1) \end{bmatrix} }_{x_d(k)} + \underbrace{ \begin{bmatrix} b_1 \\ b_2 \\ 0 \end{bmatrix} }_{\Gamma } \delta (k) \end{aligned}$$

As mentioned the $v_y$ is the output of the system, and the derivative order for the output is selected to $\nu =1$, since the lateral acceleration of the vehicle can be measured. Therefore, the output of the system in the context of the error-based ultra-local model, is selected to the lateral acceleration ($y^{\nu }_{ref}=a_y$). Thus, the output matrix is defined as: $C_d^T= [-\frac{1}{T_s}, \, v_x, \, \frac{1}{T_s}]$. Note that, for the computation of the lateral acceleration, the estimated lateral velocities are used ($\hat{v}_y$).

The computation of the nominal input signal is carried out using a deadbeat-like controller^33^. Based on the discrete state space representation and the measured lateral acceleration of the system, the nominal control input can be determined as:

23 $$\begin{aligned} u_{nom,ref}=\frac{a_{y}(k)-C_d^T \Phi (k)x_d(k)}{\Gamma (k)}. \end{aligned}$$

#### Computation of $y^{(\nu )}_{ref}$

In the next step, the computation of the reference lateral acceleration is shown. Similar to the nominal reference signal computation, the reference output of the system is derived from the nominal model and the steering angle from the previous step. Based on this nominal model, the lateral velocity for the next time step can be determined. Consequently, a derivative algorithm is applied to estimate the lateral acceleration. In this paper, the ALIEN filter algorithm is used for derivative estimation^34^. The first order derivative can be approximated. Using the Simpon’s rule for three point method:

24 $$\begin{aligned} \int _{a}^{b} f(x) \approx \frac{b-a}{6} \bigg (f(a) + 4f \big ( \frac{a+b}{2} \big ) + f(b) \bigg ). \end{aligned}$$

Applying the ALIEN filter algorithm to the estimated lateral velocity, the lateral acceleration can be computed. Finally, from ($u_{nom,ref}, y^{(\nu )}_{ref}$) the nominal ultra-local model can be determined for each time step.

### Tuning the parameter $\alpha$

The tuning of the free parameter $\alpha$ is not a straightforward task since there cannot be found ultimate solutions in the literature. In the paper, an iterative process is used to compute the optimal value for $\alpha$. More precisely, a function of $v_x$ and $\alpha$ since the optimal value can vary along with the longitudinal velocity. Firstly, several test scenarios are carried out at different longitudinal velocities, and the measurable signals are saved. A detailed description of the test vehicle and the sensor set can be found in Section 4. During the collection of the tuning dataset, the vehicle is equipped with high-precision sensors. Secondly, a set of measurements $\mathcal {A}_i=\{v_{x,i},v_{y,i},\dot{\psi }_i,\delta _i,a_{y,i}\}$ is collected. Then, the dataset is divided into subsets $\{\omega _1,\omega _2 ... \omega _n\} \subseteq \Omega$ , which are defined by a specific range of longitudinal velocity $\mathcal {A}_i\in \omega _j$ $\{v_{x,i}| v_{x,min,j}<v_{x,i}<v_{x,max,j}\}$ with upper and lower bounds: $v_{x,min,j}$ and $v_{x,max,j}$. This means that the goal is to determine the value of the tuning parameter with respect to the longitudinal velocity.

Finally, the optimization problem for the parameter $\alpha$ computation can be formed as:

25 $$\begin{aligned} \min _{\alpha _j} \sum _{i=1}^{n} (v_{y,i}-\hat{v}_{y,i})^2, \quad v_{y,i}\in \omega _j \end{aligned}$$

with the index of the subsets *j* and *i* the running variable The iteration consists of the following steps:

1. LPV-based observer design using a nominal model.
2. Initialization of the tuning parameter $(\alpha _0)$
3. Evaluate the whole test scenario.
4. Computation of the error vector between the estimated the measured lateral velocities $e_{m,i}=||v_{y,i} -\hat{v}_{y,i}||$.
5. If $\frac{1}{N}\sum _{i}^{N} e_{m,i} \ge \frac{1}{N}\sum _{i}^{N} e_{m-1,i}$ or $m>M_{max}$, quit the iteration.
6. $\alpha _m=\alpha _{m-1}+\Delta \alpha \frac{d (\frac{1}{N}\sum _{i}^{N} e_{m,i})}{d \alpha }$ then jump to Step 3.

During the operation of the observer, actual $\alpha (v)$ value is interpolated using the computed $\alpha$ values for the specific longitudinal velocities: $\alpha (v) = \alpha _{n-1} + \frac{v - v_{n-1}}{v_n - v_{n-1}} (\alpha _n - \alpha _{n-1}), \forall v \in [v_{n-1}, v_n]$.

**Pseudo code of the tuning algorithm**

**Algorithm 1:**
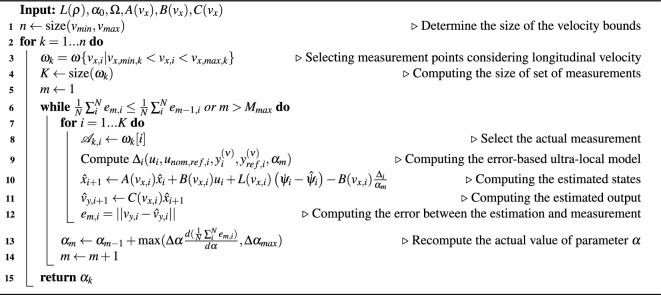
Optimization of parameter $\alpha$

The optimization process for the parameter $\alpha$ is presented in Algorithm 1. The main idea is to start with a high initial value of $\alpha$, which means, that the observer algorithm mainly uses the model-based component. In each iteration step $\alpha$ is decreased in the initial phase of the optimization. Then, close to the optimal value, the new value is recomputed with the gradient of the error. To ensure the feasibility of the algorithm, the maximum change between two $\alpha$ values is constrained. This restriction helps to avoid inappropriately low values of $\alpha$, which could lead to high oscillations. In practice, the maximum step size between iterations is set to a relatively small value. In this paper, it is limited to $\Delta \alpha _{\text {max}} = 10$. Although this results in slower convergence, the optimization is performed offline, allowing for improved robustness in the convergence process. In this paper, the initial value is set to $\alpha _0 = 200$. This approach is similar to the method described in^34^.

Application of the tuning process: The tuning algorithm uses sinusoidal-shaped reference trajectories in order to: 1.) ensure measurements are saved under steady-state conditions 2.) decrease the effect of the integration of the error signal along straight motion.

**Fig. 3:**
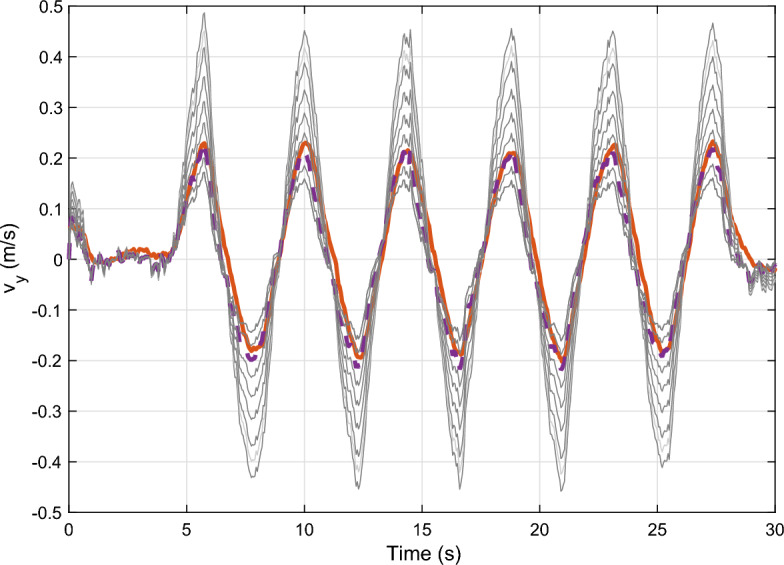
Optimization of parameter $\alpha$.

In Fig. 3 an example is shown for the optimization of the tuning parameter. The red line shows the output of the proposed observer, while the dashed purple line demonstrates the measured signal. The other gray lines show the inner steps of the optimization algorithm. As it can be seen, the optimization converges to the optimal solution.

**Fig. 4:**
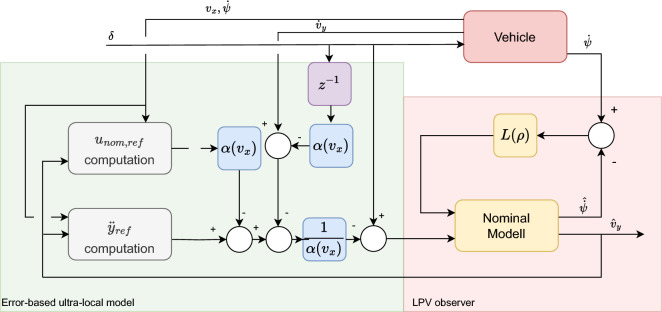
Structure of the algorithm.

The whole structure of the algorithm is illustrated for this specific application in Fig. 4.

## Validation of the proposed algorithm

In this section, the proposed observer algorithm is validated through several test scenarios. Firstly, the lateral velocity estimator is tested on real measurements, which involves general test cases such as sinusoidal steering signal, and double lane change maneuvers at different longitudinal velocities. The test vehicle was a Lexus RX450H, which was equipped with a high-precision Vectornav Dual GNSS VN-310 sensor. The parameters of the vehicle are shown in Table 1.

**Table 1:** Parameters of the test vehicle

*m*	1711 (kg)
$l_f,lr$	1.2, 1.46 (*m*)
$I_z$	2482 $(kgm^2)$
$C_f,C_r$	220000, 230000 (*N*/*rad*)

Secondly, the twin model of the vehicle has been built up and parameterized in the high-fidelity simulation software, CarMaker. After the validation of the twin model, it is used to perform an additional test scenario on a low $\mu$ surface.

### Sinusoidal steering signal

In the first case, the sinusoidal steering angle is used to validate the performance of the proposed observer. Furthermore, these measurements serve a second goal, they are used to optimize the parameter $\alpha$ presented in “Tuning the parameter α”. The results of the optimization is illustrated in Table 2.

**Table 2:** Values of parameter $\alpha$ at different $v_x$

$v_x$	$v_x=0$m/s	$v_x=5$m/s	$v_x=10$ m/s	$v_x=15$ m/s	$v_x=20$ m/s	$v_x=25$m/s	$v_x=30$m/s
$\alpha$	$\alpha =4$	$\alpha =16$	$\alpha =28$	$\alpha =41$	$\alpha =69$	$\alpha =77$	$\alpha =82$

**Fig. 5:**
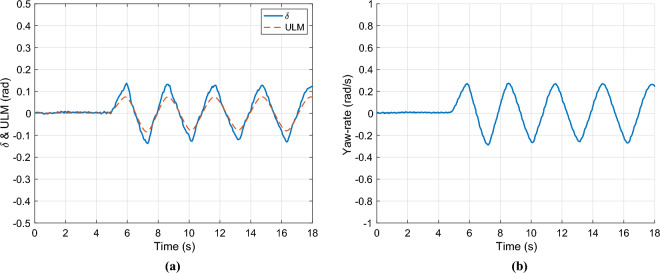
Control inputs & parameter $\alpha$.

The control input ($\delta$) and the output of the error-based ultra-local model are presented in Figure 5(a). Whilst the optimized value of the parameter $\alpha$ is illustrated in Fig. 5b

**Fig. 6:**
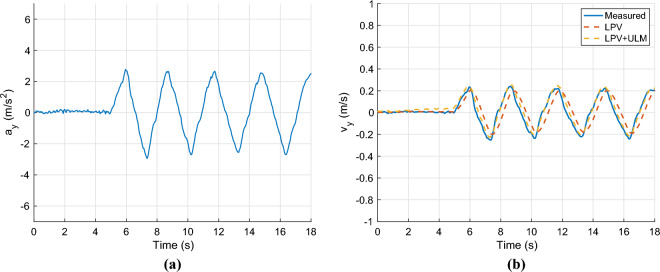
Lateral acceleration and velocity.

Finally, the lateral acceleration and velocity are depicted in Figures 6. As the second figure shows two observer algorithms are used to estimate the measured lateral velocity. The first one is a nominal LPV-based observer, which has a scheduling parameter: $v_x$, and the second one is the proposed one, which includes the error-based ultra-local as shown in the structure diagram 1. In this case, both solutions provide accurate results regarding the amplitudes of the signals, However, the nominal observer has a significant time delay of 0.2*s*. This phenomenon is caused by the steering system, which is an unmodelled dynamics of the system. Since the road wheel angle is directly computed from the steering wheel angle. The proposed algorithm is able to deal with this problem.

### Double lane change

**Fig. 7:**
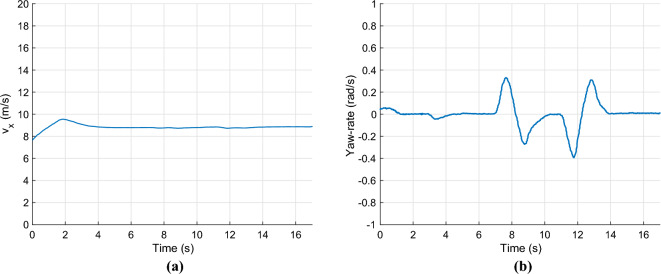
Longitudinal velocity & yaw-rate.

In the second scenario, a high lateral velocity maneuver is performed, namely a double lane change maneuver. Figure 7 shows the longitudinal velocity, which is also set to $v_x=9m/s$ in this case, and the yaw rate of the vehicle during the maneuver.

**Fig. 8:**
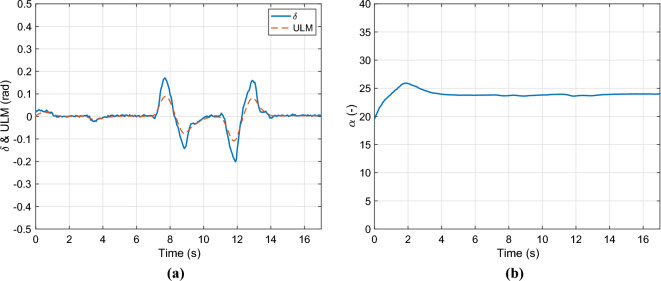
Control inputs & $$\alpha$$.

The control signals and the parameter $\alpha$ are shown in Fig. 8.

**Fig. 9:**
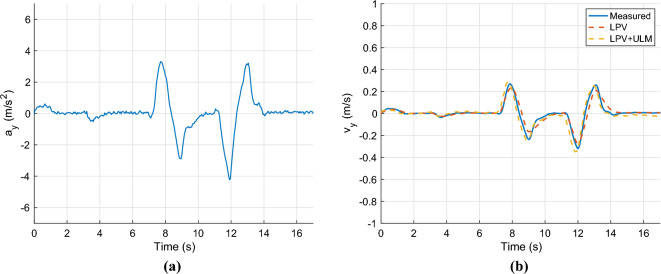
Lateral acceleration and velocity.

Finally, Fig. 9(a) presents the lateral acceleration, whose peak value is around $4m/s^2$. The estimated lateral velocities are depicted in Fig. 9b. Similarly to the sinusoidal steering signal, both solutions provide acceptable results. However, the proposed algorithm has a slightly better fit. In the next case, a more complex test scenario is presented to show the performance of the proposed observer at varying longitudinal velocities and under different conditions.

### Complex test scenario

This test serves the purposes: First, to show the effectiveness and the operation of the proposed algorithm through a complex test scenario and second to validate the twin model, which has been built up in the CarMaker simulation software.

**Fig. 10:**
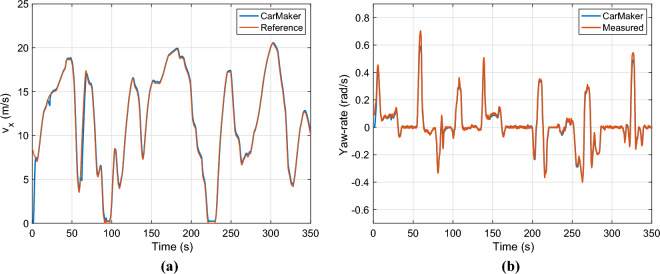
Longitudinal velocity & yaw-rate.

The velocity profile of the vehicle is presented in Fig. 10a. The velocity profile has a wide range, it varies between 0 and 20*m*/*s*. Figure 10b shows the measured and computed yaw rate of the vehicle from the CarMaker simulation software. The computed yaw-rate covers the measured one, except at a high value e.g. around 60*s*, where the measured yaw-rate reaches 0.7*rad*/*s* due to the low longitudinal velocity 4*m*/*s*. In other cases, the error is below $1\%$.

**Fig. 11:**
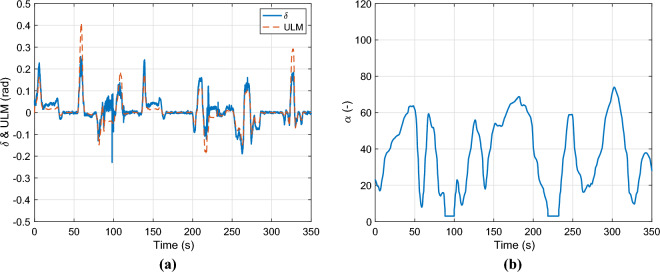
Control inputs & $$\alpha$$.

Figure 11a demonstrates the applied steering angle and the output of the error-based ultra-local model. As can be seen, when the longitudinal velocity is low, the output of the error-based ultra-local model exceeds the steering angle to compensate for the unmodelled dynamics of the system. The parameter $\alpha$ is depicted in Fig. 11b, it varies by the longitudinal velocity.

**Fig. 12:**
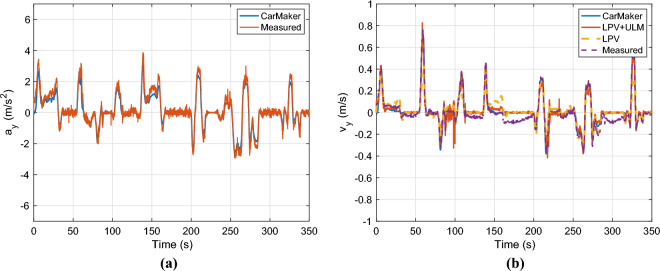
Lateral acceleration and velocity.

Finally, the lateral acceleration and velocities are presented in Fig. 12. The computed lateral acceleration covers the measured one but it has a slightly larger error than in the case of the yaw-rate. The maximal deviation is around $0.2m/s^2$. The last figure illustrates the lateral velocities for all cases. The computed yaw rate of the measured one fit well, although the measured one has a significant bias around zero lateral velocity. The nominal LPV observer provides a good solution at lower $v_y$, however at high values, it has a large error around $45\%$. The proposed observer works well in the whole dynamical range.

### Quantitative analysis

This subsection presents a quantitative analysis of the proposed combined observer design. The performance of the method is compared with two solutions: a neural network-based solution and a purely LPV-based approach. The machine learning-based solution employs a Feedforward Neural Network (FFNN) with two hidden layers consisting of 250 and 100 neurons, respectively. Moreover, Rectified Linear Unit (ReLU) functions are used as the activation functions. Further details regarding the training and implementation of the neural network can be found in^14^.

**Fig. 13:**
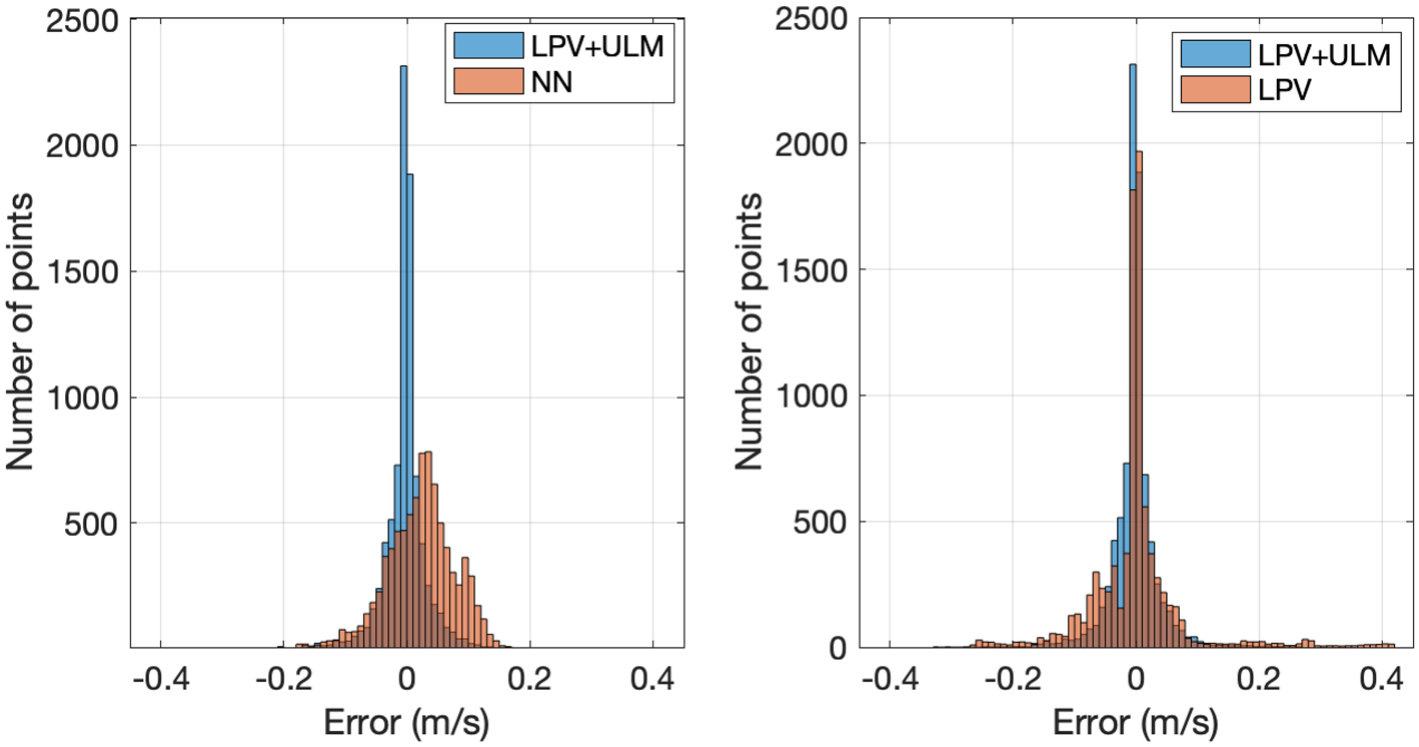
Error histogram

In Fig. 13 the histograms of the computed errors are depicted. It can be observed that the proposed method provides the most accurate results compared to the purely LPV-based approach and the machine learning-based solution. In the machine learning-based approach, a slight bias can be observed, and a higher standard deviation is also noticeable. Moreover, in the following Table, the computed errors are also presented, such as the Standard deviation (Std.), the maximum value of the error (Max.), and the sum of the Mean Squared Error (MSE). Additionally, to avoid error values, which occur along straight lines, the measured points are selected where the measured lateral velocity exceeds $|v_y| > 0.05$.

**Table 3:** Comparison of estimation errors of various observers (nominal values)

	MSE	Std.	Max.	MSE (Selected)	Std. (Selected)	Max. (Selected)
LPV+ULM	0.0219	0.0366	0.1911	0.0438	0.0542	0.1911
LPV	0.0418	0.0761	0.4182	0.0815	0.1161	0.4182
NN	0.0461	0.0603	0.2712	0.0559	0.0653	0.2712

To present the performance levels in a more comprehensible form, Table 4 shows the accuracy rate compared to the combined method, which integrates the LPV and ultra-local model-based solutions.

**Table 4:** Comparison of estimation errors of various observers (percentages)

	MSE	Std.	Max.	MSE (Selected)	Std. (Selected)	Max. (Selected)
LPV+ULM	100%	100%	100%	100 %	100 %	100 %
LPV	191%	207 %	218 %	186 %	214 %	218 %
NN	210%	164 %	141 %	127 %	120 %	141 %

Based on the results in Table 3 and Table 4, it can be concluded that the proposed observer algorithm outperforms both the machine learning-based and the classical observer methods. When the evaluation is performed using the entire dataset, the Mean Squared Error is reduced by approximately 50 % through the use of the error-based ultra-local model extension. Within the selected region, the error is reduced by 21 % compared to the machine learning-based approach. Moreover, when accuracy is compared to the LPV-based observer method, the error reduction again nearly reaches 50 %.

**Fig. 14:**
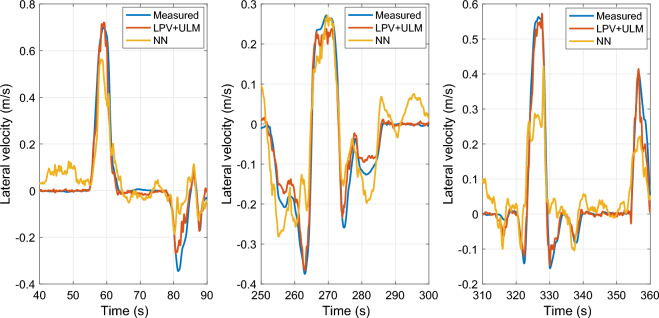
Comparison of NN and ULM approaches.

In Figure 14, selected regions of the entire simulation scenario are highlighted for comparative analysis. It can be seen that the proposed method demonstrates significantly higher accuracy than the machine learning-based approach. Moreover, the estimation provided by the neural network shows significant inaccuracies along straight trajectories. This phenomenon can be explained by the fact that measurements may not return to zero during maneuvers along straight lines. Additionally, the presence of measurement noise can highly affect the performance of the neural network-based estimation. These findings underline the importance of high-quality training datasets for machine learning methods. However, measuring lateral velocity accurately is challenging, suggesting that post-processing is a crucial step following data measurement. This additional step can significantly increase the complexity of the overall design process. Moreover, system parameters can vary over time, which affects the performance of a neural network that was trained on prior data.

On the other hand, in the error-based ultra-local model-based approach, the measured data points are not directly used for training purposes. Instead, measurements are used solely for determining the tuning parameter ($\alpha$), which suppresses the impact of measurement errors and noises on the output. This property increases the usability of the proposed method in real-world applications compared to purely machine learning-based approaches.

### Low $\mu$ scenario

**Fig. 15:**
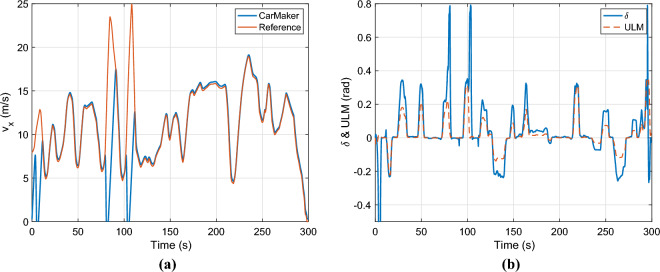
Longitudinal velocity & control inputs.

In the last scenario, the twin model and the observers are compared at a low adhesion coefficient ($\mu =0.4$). The velocity profile is shown in Fig. 15a. As can be seen, the CarMaker model cannot track the profile at high longitudinal velocities due to the low adhesion coefficient. The yaw rate of carmaker is presented in Fig. 15b, it reaches extreme values around 1.7rad/s when the longitudinal velocity is close to zero. Under these circumstances, the model-based observer fails to estimate the lateral velocity.

**Fig. 16:**
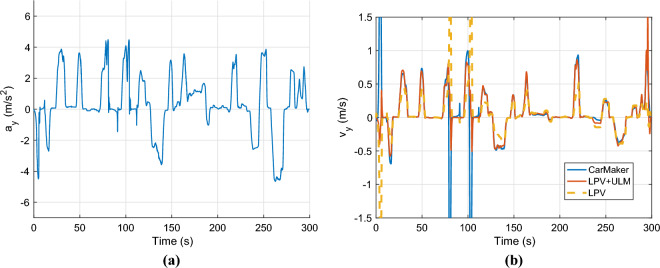
Lateral acceleration and velocity.

The control inputs and the parameter $\alpha$ are shown in Fig. 15b. The lateral acceleration is illustrated in Fig. 16a. The maximum of it is around $4.4m/s^2$, which is the physical limit of the vehicle at this $\mu$ value. Finally, the lateral velocity is presented in Fig. 6b. The nominal LPV observer cannot deal with the low $\mu$ surface it has a significant error throughout the simulation. However, the performance of the proposed method remains high. The maximal error in the cases, when the velocity is higher than 5m/s the error peaks at 0.2 m/s.

The only relevant estimation error can be observed at low lateral velocity ($v_y$) e.g. between $t=230-240s$. It is caused by the low lateral acceleration ($a_y$) since the error-based ultra-local model doesn’t get enough excitations to determine the error between the real system and the nominal model. This phenomenon only appears at low lateral velocity thus it does not have a high impact on the overall results. Another limitation of the proposed algorithm lies in the tuning of parameter $\alpha$. It must be recomputed for all specific vehicles and maybe for all possible conditions. Although the tuned parameter $\alpha$ worked under all presented circumstances, higher accuracy can be achieved by refining this parameter.

## Conclusion

In the paper, a new combined observer design method has been presented for estimating the lateral velocity of ground vehicles. The proposed method was based on the LPV framework and the error-based ultra-local model. The ultra-local model was used to capture the nonlinear, and unmodeled dynamics of the system. There was one additional signal to be measured compared to the conventional LPV observer: lateral acceleration. The operation and the effectiveness of the proposed algorithm have been tested: 1. in a high fidelity vehicle dynamics simulation software, CarMaker, 2. using real test measurements from a Lexus RX450h with a high precision GPS. The simulations have shown that the algorithm could work under various circumstances such as at low adhesion coefficient and in a wide range of longitudinal speed. The real test measurements showed that the combined observer was able to provide the accuracy of the high-precision GPS, and even give better results at low lateral velocity. Regarding the limitations of the algorithm, two main issues can be highlighted. First, at low lateral acceleration, the error-based ultra-local model cannot provide high-accuracy results due to the lack of excitation. Second, the recomputation of the free parameter of the error-based ultra-local model ($\alpha$) may increase the accuracy of the proposed observer for each different test case (e.g. low adhesion coefficient surface). The future research topic is to combine the presented observer design approach with the ultra-local model-based controller to provide a co-design method, which can make the process faster and more efficient.

## Data Availability

All data generated or analyzed during this study is available from the corresponding author upon reasonable request.
